# Editorial: A home for virology, ecology, epidemiology, and evolutionary biology

**DOI:** 10.1093/ve/vev001

**Published:** 2015-03-26

**Authors:** Santiago F. Elena, Oliver G. Pybus

**Affiliations:** ^1^Instituto de Biología Molecular y Celular de Plantas, CSIC-UPV, València, Spain, ^2^The Santa Fe Institute, Santa Fe, New Mexico, USA and ^3^Department of Zoology, University of Oxford, UK

Viruses make headline news on an almost daily basis. Sometimes the news is positive, a report on the development of new anti-viral drugs or a reduction in transmission, perhaps. However, often the story will relate to a gloomier theme, for example, the appearance of new viral epidemics, the evolution of drug resistance, or falling vaccine coverage. The appearance of Ebola virus in West Africa since 2014 represents just the latest of a long series of devastating viruses that have emerged or expanded in humans in recent years, including Middle East Respiratory Syndrome (MERS) and Severe Acute Respiratory Syndrome (SARS) coronaviruses, Chikungunya virus, highly pathogenic avian influenza viruses, West Nile virus, and various human enteroviruses, and bunyaviruses. This list is both selective and anthropocentric and excludes numerous new epidemics in livestock (e.g., Schmallenberg virus), crop (e.g., tomato torrado virus), and wild animal populations (e.g., phocine distemper virus). The impacts of viral epidemics may extend beyond death and illness to cause substantial economic losses and social instability. Such effects are not limited to new or exotic viruses, as established and well-characterized viral diseases persist despite tremendous efforts to control and eradicate them. Important pathogens in this category include HIV/AIDS, human influenza viruses, dengue viruses, hepatitis viruses, human papillomaviruses, and rabies virus.

One reason that viruses are such potent adversaries is their great potential for genetic diversity and evolvability, a characteristic that they owe to a combination of short generation times, very large population sizes, and (in some but not all instances) error-prone replication mechanisms. Strains that escape host immune responses, are resistant to antiviral drugs, or, in the case of plant viruses, that break transgenic or natural genetic resistance, may arise in a viral population soon after it is challenged by the corresponding antiviral measure. Together, the outlook is mixed: the perspectives for future eradication or control are balanced by the emergence or re-emergence of new foes.

Although genetic diversity is an essential part of virus biology, classical approaches to virus control often ignore evolutionary processes and focus on understanding in great detail the molecular bases of pathogenesis, virus–host interaction, and drug–virus interference. This is despite the fact that evolutionary concepts are key to the correct interpretation of molecular variation among virus strains. Some experimental biologists, on the other hand, have taken advantage of the rapid evolution of many viruses and chosen to use them as model organisms for the investigation of fundamental questions in evolutionary biology (e.g., Elena and Sanjuán 2007). Moreover, in recent years, virus epidemiologists have begun to exploit the increasing availability of virus genome sequences and incorporated evolutionary thinking in their approach, leading to new insights into the ecological origins and transmission of viruses (e.g., Holmes 2009; Pybus and Rambaut 2009). Lastly, computational and theoretical biologists are developing increasingly complex models of virus behavior across all biological scales, from the cellular to the global, many of which attempt to explicitly represent the generation and dynamics of viral genetic diversity under different conditions (e.g., Nowak and May 2000; Wilke 2003; Luksza and Lässig 2014). Unfortunately, it is rare for all these diverse approaches to be taken into account when new strategies for virus control are designed. Why? Perhaps, at least until recently, there was insufficient exchange of ideas and concepts among these disciplines.

**Figure 1.**
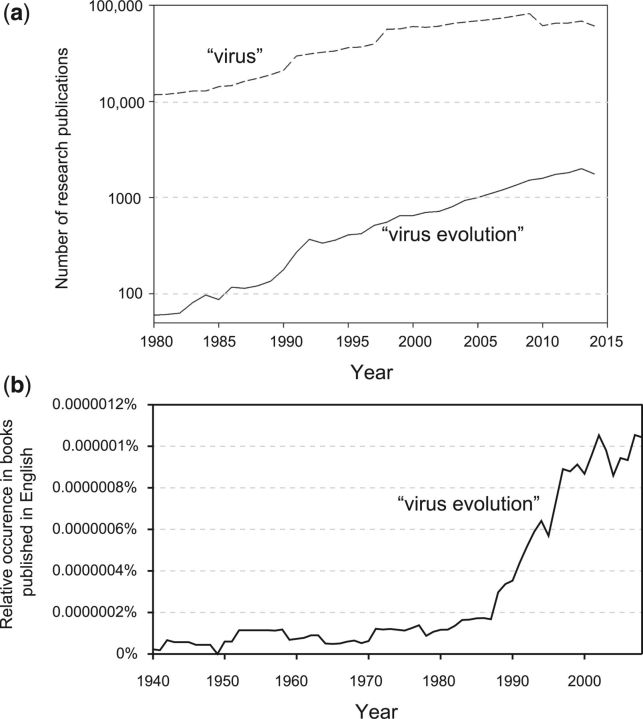
(a) Number of scientific articles using the terms ‘virus evolution’ or ‘virus’ in Thomson Reuters Web of Science between 1980 and 2015. Solid line: topic = (virus evolution) OR title = (virus evolution). Dashed line: topic = (virus) OR title = (virus), subtracting the numbers shown in the solid line. (b) Relative frequency of the term ‘virus evolution’ in the corpus of books published in English between 1940 and 2008. Data from Google’s Ngram Viewer (https://books.google.com/ngrams).

The study of virus evolution in its own right has flourished in the last 25 years and the subject undoubtedly gained greater recognition within the biological sciences after the discovery that evolution is an essential component of HIV infection. Since then, the number of published articles that use the term ‘virus evolution’ in the article title or summary has grown exponentially and doubled every 6–7 years (Fig. 1a). An equally striking pattern is seen if we plot occurrence of the same term in books written in English (Fig. 1b). The rate of growth of scientific papers that include ‘virus evolution’ is nearly twice that of those that mention only ‘virus’ (Fig. 1a) and approximately three times faster than the growth rate of PubMed as a whole (Lu 2011).

Where do all these manuscripts get published? Despite an explosion in virus evolution research activity, publications on the topic are scattered among a large number of journals that belong to a variety of categories from the Institute of Scientific Information (ISI). Although many studies appear in evolutionary biology journals, particularly those on viral experimental evolution, mathematical modeling, molecular evolution, and phylogenetics, a large proportion are submitted to journals that focus on virology and pathogenesis. In these disciplines, some editors express a preference for ‘mechanistic’ studies within a clear hypothetico-deductive framework and may not appreciate the importance of inferential and observational work within population and evolutionary biology. Further, several virology journals focus on either animal and plant viruses, so that relevant articles may not come to the attention of researchers from the other field. Viruses of bacteria, archaea, fungi, and protists are served comparatively poorly by the current literature, yet these groups are very likely to comprise the majority of viral genetic diversity on Earth. To add further fragmentation, some important theoretical work on virus variability and evolution is published in specialized mathematical journals that will not be well known to laboratory and field researchers.

We believe that the study of virus evolution would benefit from a common forum in which findings and ideas can be shared. We have established the journal *Virus Evolution* with this aim in mind, and we hope that it will grow into a successful and dynamic inter-disciplinary community of researchers interested in understanding why and how viruses have and continue to evolve. We aim to cover all aspects of virus evolution, ecology, and diversity with no restriction on host range or research methodology. To achieve this goal, we have assembled an Editorial Board whose members have made many important contributions to the field. The Board has expertise in animal, plant, and bacterial viruses and in a wide range of techniques, including experimental evolutionary biology, molecular epidemiology, metagenomics, structural biology, population genetics, ecology, and molecular virology. One benefit of a focused journal, such as *Virus Evolution,* is that the Editorial Board shares with the authorship a passion for the subject.

Our editorial philosophy is that *Virus Evolution* exists first and foremost to serve its authors and readers. To make publication in the journal a more pleasant experience, we impose no specific formatting requirements at submission: the manuscript can be provided in any style so long as it is readable by reviewers. Standard formatting will be requested only ‘after’ a paper has been accepted, at which point it should seem less of a chore. We will operate a traditional peer-review process but one that will emphasize the quality of reviews as well as their speed. Published papers will be available to read by all under an Open Access model that is compliant with all major funding bodies including the USA National Institutes of Health and UK Wellcome Trust. Lastly, accepted manuscripts will be visible online in the shortest possible time after acceptance.

We very much look forward to receiving your submissions and to begin working with the community to make *Virus Evolution* a vibrant and successful home for your research.
